# Combined hearing and vision screening programs: A scoping review

**DOI:** 10.3389/fpubh.2023.1119851

**Published:** 2023-03-14

**Authors:** Ilze Oosthuizen, Caitlin Frisby, Shelly Chadha, Vinaya Manchaiah, De Wet Swanepoel

**Affiliations:** ^1^Department of Speech-Language Pathology and Audiology, University of Pretoria, Pretoria, South Africa; ^2^Virtual Hearing Lab, Collaborative Initiative Between University of Colorado School of Medicine, Aurora, CO, United States; ^3^University of Pretoria, Pretoria, South Africa; ^4^WHO Programme for Prevention of Deafness and Hearing Loss, World Health Organization, Geneva, Switzerland; ^5^Department of Otolaryngology-Head and Neck Surgery, University of Colorado School of Medicine, Aurora, CO, United States; ^6^UCHealth Hearing and Balance, University of Colorado Hospital, Aurora, CO, United States; ^7^Department of Speech and Hearing, School of Allied Health Sciences, Manipal Academy of Higher Education, Manipal, India; ^8^Ear Science Institute Australia, Subiaco, WA, Australia

**Keywords:** hearing impairment, vision impairment, combined sensory screening, hearing screening, vision screening, health screening, low-and middle-income countries

## Abstract

**Background and aim:**

The World Health Organization (WHO) estimates that 1.5 billion and 2.2 billion people have hearing and vision impairment, respectively. The burden of these non-communicable diseases is highest in low- and middle-income countries due to a lack of services and health professionals. The WHO has recommended universal health coverage and integrated service delivery to improve ear and eye care services. This scoping review describes the evidence for combined hearing and vision screening programs.

**Method:**

A keyword search of three electronic databases, namely Scopus, MEDLINE (PubMed), and Web of Science, was conducted, resulting in 219 results. After removing duplicates and screening based on eligibility criteria, data were extracted from 19 included studies. The Joanna Briggs Institute Reviewer Manual and the Preferred Reporting Items for Systematic Reviews and Meta-analyzes (PRISMA) Extension for Scoping Reviews were followed. A narrative synthesis was conducted.

**Results:**

Most studies (63.2%) were from high-income countries, with 31.6% from middle-income and 5.2% from low-income countries. The majority of studies (78.9%) involved children and the four studies reporting on adults all included adults above 50 years of age. Vision screening was most commonly performed with the “Tumbling E” and “Snellen Chart,” while hearing was typically screened using pure tone audiometry. Studies reported referral rates as the most common outcome with sensitivity and specificity rates not reported in any included articles. Reported benefits of combined vision and hearing screenings included earlier detection of vision and hearing difficulties to support functioning and quality of life as well as resource sharing for reduced costs. Challenges to combined screening included ineffective follow-up systems, management of test equipment, and monitoring of screening personnel.

**Conclusions:**

There is limited research evidence for combined hearing and vision screening programs. Although potential benefits are demonstrated, especially for mHealth-supported programs in communities, more feasibility and implementation research are required, particularly in low- and middle-income countries and across all age groups. Developing universal, standardized reporting guidelines for combined sensory screening programs is recommended to enhance the standardization and effectiveness of combined sensory screening programs.

## Introduction

Hearing and vision impairments are two of the most common non-communicable conditions with global estimates of 1.5 and 2.2 billion affected people, respectively (1, 2). The global annual costs associated with unaddressed hearing and vision impairment amount to over 980 and 24.8 billion USD, respectively (1–3). The prevalence is estimated to increase due to population growth, an aging population, and lifestyle changes linked to these impairments (1). Although these impairments are evident globally, both hearing and vision impairment are four times higher in low- and middle-income countries (LMICs) than in high-income countries (4–6). These disparities across income groups can be attributed to limited access to healthcare or unavailable healthcare in LMICs (2). This is due to a severe shortage and disparate rural/urban distribution of trained healthcare professionals, infrastructure, and resources, and the high costs associated with traditional hearing and vision healthcare (1, 2, 7–10).

Approximately 50 to 60% of hearing or vision impairments could be prevented or corrected (1, 2). This emphasizes the importance of periodic hearing and vision screenings throughout the life course. A recent study (11) highlighted that identifying hearing impairment in children does not necessarily predispose to or exclude vision impairment. Periodic screening could allow for early identification to minimize or negate the negative implications of hearing and vision loss on early childhood development and education. Sensory input from both hearing and vision is key to optimal learning outcomes (2, 12, 13). In adults, timely treatment through early detection can also support improved employment opportunities and active participation in the economy with a wider impact on society (1–3, 12, 14, 15). Considering the widespread prevalence of sensory impairments across the life course, with 9 to 28% of adults over the age of 70 years estimated to have both a hearing and vision impairment, also emphasizes the importance of periodic screening (16, 17). A combined sensory impairment in adults has been linked to decreased quality of life compared to individuals with only one sensory impairment and an increased risk of falling, depression, and even mortality (18).

Combined hearing and vision screening programs are potentially more cost-effective and therefore could enable widespread screening, particularly in LMICs with limited resources. A combined service can extend the value of available resources as the population groups most affected by hearing problems are often the same as those with the highest burden of vision problems, i.e., older adults and children of school-going age. Furthermore, using the same screening personnel may reduce time, and associated costs as screeners can receive combined training and share resources (19). Recent studies have demonstrated that identification and diagnosis of hearing and vision impairments is possible in community-based programs (11, 20). Furthermore, training of non-professional community healthcare workers to facilitate identification and primary care for both conditions has been successfully implemented (11, 21–23). Integration of hearing care into existing eye care programs has also been demonstrated in South-East Asia under the Sound Hearing 2030 program (24). In light of the World Health Organization (WHO) recommendation of universal health coverage and integrated service delivery to improve ear and eye care services (1, 2), the current evidence for combined hearing and vision screening programs should be explored. This scoping review, therefore, aimed to identify and describe the published evidence for combined hearing and vision screening programs.

## Methods

This scoping review was guided by the Joanna Briggs Institute Reviewer Manual (25) and the Reporting Items for Systematic Reviews and Meta-Analyzes Extension for Scoping Reviews (PRISMA-ScR) checklist (25) (Supplementary Material 1).

### Eligibility criteria

The literature search was conducted during the second quarter of 2022, and the final search was conducted on 14 June 2022. Eligibility criteria were guided by the Population, Concept, and Context (PCC) framework stipulated in the Joanna Briggs Institute Reviewer Manual (25) as outlined in Table 1. Articles had to be empirical and published in English-language peer-reviewed journals to be included in this review. No time, age, or geographic restrictions were made. All publications examining combined vision and hearing screening programs were included. Non-peer-reviewed publications, reviews, discussion papers, dissertations/ thesis, conference papers, opinions, viewpoints, and pre-prints were excluded. Any studies where either vision or hearing screening was conducted alone were excluded.

**Table 1:** Eligibility criteria using the population, concept, and context (PCC) criteria.

**Study domain**	**Inclusion criteria**	**Exclusion criteria**
Population	No age restriction	No exclusions
Concept	Combined vision and hearing screening programs	Studies focusing on either vision or hearing screening alone
Context	Open: No exclusion of settings. Could include healthcare (public or private), educational, or community-based settings	No exclusions

### Information sources and search strategy

A keyword search was conducted on three electronic databases (Scopus, Web of Science, and MEDLINE[PubMed]). These databases have also been used for reviews in both hearing and vision studies recently (26–30). An exploratory search utilizing keywords (“hearing screening”) AND (“vision screening”) was conducted on 17 May 2022 on Scopus. The keyword search was then expanded to include (“hearing test” OR “hearing screening”) AND (“vision screening” OR “eye test”). Keywords were expanded a final time, and the final keyword search strategy included (“hearing test” OR “hearing screening”) AND (“vision screening” OR “eye test” OR “visual acuity”). The final keyword search was conducted across all three databases. The references cited in the included articles were also hand searched to identify possible additional studies.

### Data charting and data items

Articles meeting the inclusion criteria were screened for duplicates using an online review tool, Rayyan software [https://www.rayyan.ai/; (31)]. Any duplicate articles were removed. The first (IO) and second (CF) authors screened the identified articles' titles, abstracts, and full text. The first (IO) and second (CF) authors cross-checked 50% of each other's screening of the identified articles and confirmed that the exclusion criteria were applied consistently. Data extraction from the included studies was conducted by the first (IO) and second (CF) authors. The remaining authors (SC, VM & DS) cross-checked the data extraction. Data were extracted onto a Microsoft Excel spreadsheet (Supplementary Material 2). The following data were extracted if reported: author details, publication year, primary database, study design, population, sample size, setting, the country conducted in, income bracket of country, type of tests conducted, the device(s) operated on, facilitators or test personnel, referral criteria, referral rates, follow-up rates, program cost, test duration, sensitivity, specificity, benefits of combined screening, and challenges to combined screening.

### Synthesis of results

The findings were synthesized with input from all authors to describe the characteristics of combined vision and hearing screening programs, the most frequently reported referral criteria, and the outcomes of such screening programs. Furthermore, the clinical implications regarding reported benefits and challenges and recommendations for future implementations were summarized. Due to the heterogeneity of the studies included and the scoping nature of the review, results are presented descriptively. Descriptive statistics were utilized to analyze the data from included articles on SPSS (version 27.0; IBM Corp). Studies that used more than one type of test, facilitator, setting, or population group were counted more than once within each relevant section. The WHO classification was used to analyze articles according to income classification (32).

## Results

### Search results

**Figure 1:**
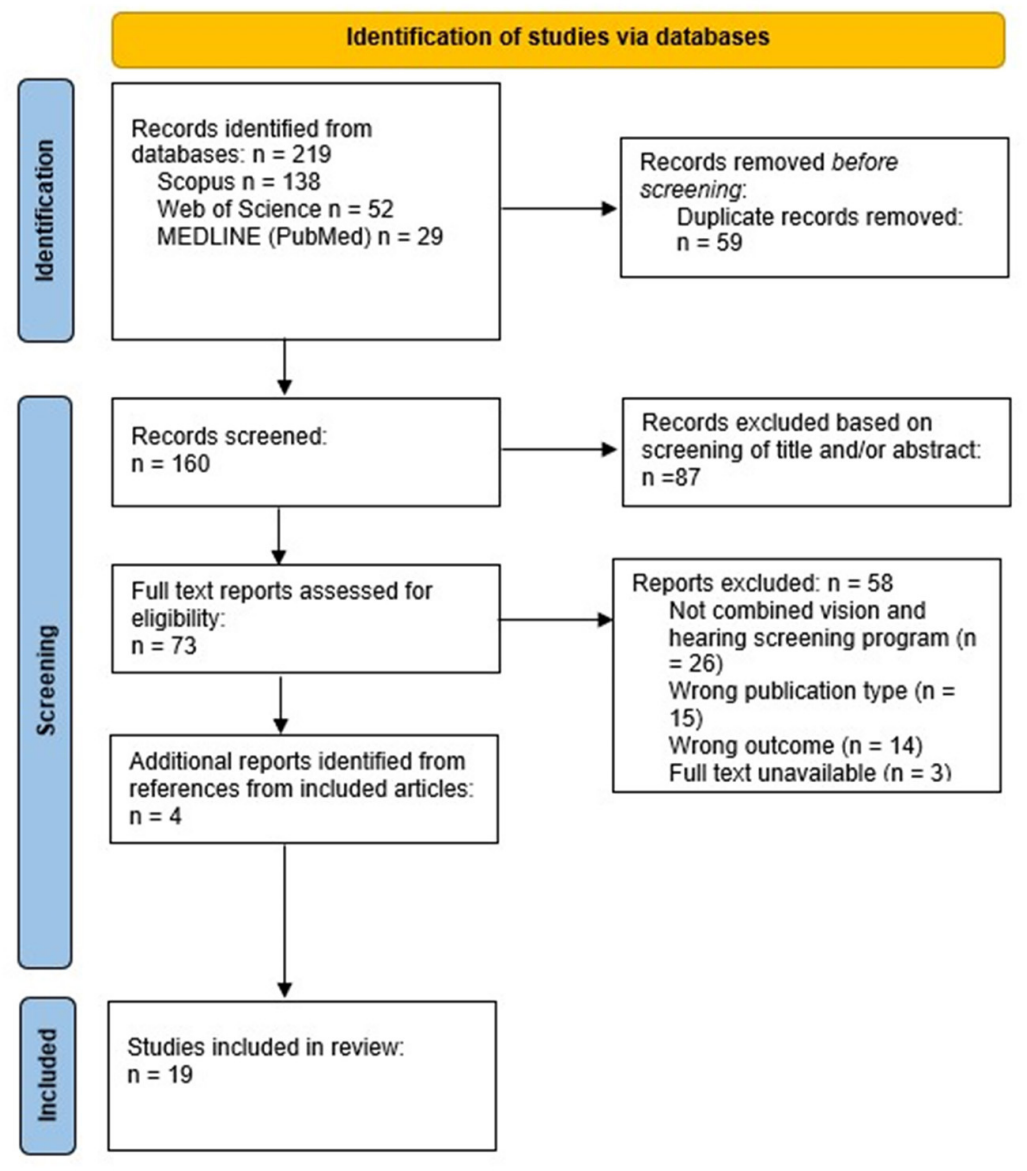
PRISMA flow diagram details the search and selection process applied during the scoping review.

From the 219 unique records identified in the searches, 15 studies were deemed appropriate for inclusion (Figure 1). The references of the included articles were hand searched and reviewed, and an additional 4 (21.1%) articles were included. A total of 19 unique articles were identified and included in this review. The initial search was conducted on Scopus and identified 11 (57.9%) of the included articles. MEDLINE (PubMed) and Web of Science were then searched and identified 3 (15.8%) and 1 (5.2%) additional articles, respectively, that were included. The PRISMA flow diagram that details the search and selection process applied during the scoping review is shown in Figure 1.

### Characteristics of the included studies

**Figure 2:**
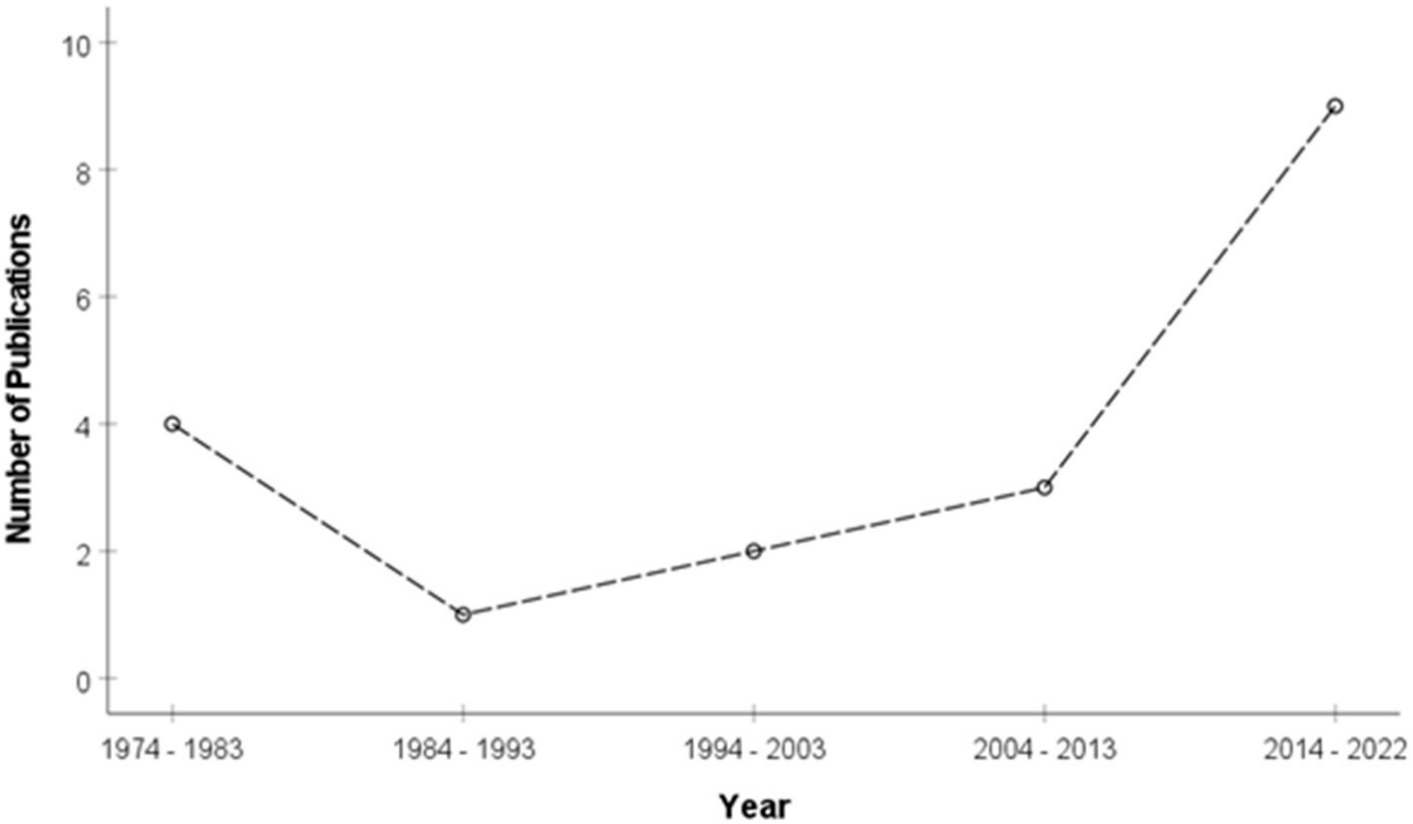
Combined hearing and vision screening studies (*n* = 19) published between 1974 and 2022.

All articles included in this review were published between 1974 and 2022 (Figure 2). Vision and hearing screenings in the included studies were conducted in 8 unique countries, including the United States of America (*n* = 5), South Africa (*n* = 4), Australia (*n* = 4), Sweden (*n* = 2), Italy (*n* = 1), New Zealand (*n* = 1), Mexico (*n* = 1), Malawi (*n* = 1), and India (*n* = 1). Studies were primarily conducted in high-income countries (63.2%; n = 12), with 31.6% (*n* = 6) and 5.2% (*n* = 1) conducted in middle-income and low-income countries, respectively. However, one high-income country and three middle-income country studies were conducted in low-income communities. The majority of the studies (73.7%; n = 14) specified the study design followed. In terms of the level of evidence provided by different research designs (33), a few cross-sectional and cohort studies were included but no included studies were of a higher design (e.g., randomized control trials). All studies conducted the combined screening on the same individuals, and most studies (78.9%) involved children (see Table 2). The median sample size of all included articles was 695 participants.

**Table 2:** Characteristics of identified studies (*n* = 19) on combined hearing and vision screening programs across population, testing type, and setting.

	**% (*n*)**	**Sample size (range)**
**Population**		
Adults (aged 50–93)	21.1 (4)	49–1,511
Children (aged 0–18)	78.9 (15)	31–762 627
< 3 years^*^	21.1 (4)	
3–7 years^*^	52.6 (10)	
>7 years^*^	21.1 (4)	
**Setting** ^**^		
Schools	57.9 (11)	132–254,725
Homes	21.1 (4)	235–1,511
Hospital	10.5 (2)	31–762,627
Park	5.2 (1)	741
Van	5.2 (1)	442
Special care unit	5.2 (1)	49
University	5.2 (1)	1,330
Community-based unspecified	5.2 (1)	841

* Ages of participants in included articles ranged across the age groups and were counted once within each relevant group.

** Total number of articles shown is 22, as three studies were conducted in multiple settings.

### Vision screening

The most commonly used vision tests were the “Tumbling E” and “Snellen Chart,” reported in 18.4 and 15.8% of studies, respectively (Table 3). Facilitators for vision screening tests varied from vision technicians to medical specialists (e.g., pediatrician, ophthalmologist), with community health workers (CHWs) and nurses (including nursing students, nurses, and school nurses) used most often to conduct the screening (14/19). Specifically, 31.6 and 47.4% of the studies used CHWs and nurses, respectively, to facilitate vision screening tests. Referral for further visual assessment was based mainly on a visual acuity score obtained from several screening tests, including the Tumbling E, Snellen chart, linear E-chart (Oculus), modified parr-letter-match, Sheridan Gardner charts, PattiPics chart, and Precision Vision (Table 3). For the pediatric population, the referral visual acuity score ranged from less than 6/6 (i.e., 0.0 logMAR) to 6/18 in one or both eyes. However, a visual acuity score of 6/12 (i.e., 20/40 or 0.3 logMAR) was used most frequently (*n* = 5). For the adult population, a visual acuity score of worse than 6/12 or worse than 20/60 was reported as referral criteria (*n* = 2). The red reflex test was specifically used in the newborn population with a referral criterion when no red and identical reflection in both eyes were obtained (*n* = 1). More than half of the visual screening tests did not specify referral criteria.

**Table 3:** Vision screening test type (*n* = 38), facilitators and referral criteria used across studies (*n* = 19).

Type of test: vision screening^*^	**% (*n*)**	**Facilitator type (*n*)**	**Referral criteria**
Tumbling E	18.4 (7)	Community healthcare workers (4) Trained and standardized interviewers (1) Medical doctor (1) Vision consultant (1) Nurse (2)	*Children:* Visual acuity of < 0.3 LogMAR in both eyes or < 0.4 LogMAR in one eye (*n*=3) Visual acuity worse than 20/30 (preschool) or worse than 20/20 (older child) in each eye (*n*=1) *Adults:* Visual acuity worse than 20/60 (*n* = 1) Visual acuity < 6/12 (*n* = 1)
Snellen chart	15.8 (6)	Nurses (3) Nursing students (1) Community healthcare workers (1) Doctors (3) Medical students (1) Eye technicians (1)	*Children:* Visual acuity < 1.0 (6/6) in one or both eyes (*n* = 1) *Adults:* Remedial abnormality present (*n* = 1)
Red reflex	5.3 (2)	Nursing students (1) Pediatricians (1) Ophthalmologists (1)	*Newborns:* No red reflection and reflection not identical in both eyes (*n* = 1)
Cover test	5.3 (2)	Community healthcare workers (1) Nursing students (1)	*Children:* Large amount of movement or turned eye (*n* = 1)
Linear E-chart (Oculus)	2.6 (1)	School nurse (1)	*Children:* Visual acuity ≤ 0.9 in one or both eyes (*n* = 1)
Modified Parr letter-matched	2.6 (1)	Nurses (1) Vision technicians (1)	*Children:* Visual acuity of 6/12 or lower in one or both eyes (*n* = 1)
Sheridan Gardner charts	2.6 (1)	Nurses (1) Vision technicians (1)	*Children:* Visual acuity of 6/12 or lower in one or both eyes (*n* = 1)
PattiPics Chart	2.6 (1)	Community healthcare workers (1)	*Children:* Visual acuity 6/9–6/18 or worse (*n* = 1)
Precision Vision	2.6 (1)	Community healthcare workers (1)	*Children:* Visual acuity 6/9–6/18 or worse (*n* = 1)
Glazed flippers þ 1.50 (Cyclopean Design)	2.6 (1)	Community healthcare workers (1)	*Children:* Eyes misalign through all directions of gaze (*n* = 1)
Color vision test (Waggoner).	2.6 (1)	Community healthcare workers (1)	*Children:*> 3 errors
Animal chart	2.6 (1)	Nursing students (1)	NR
Parental Questionnaire	2.6 (1)	Nursing students (1)	NR
Lea visual acuity test	2.6 (1)	Nursing students (1)	NR
Random Dot E stereopsis test.	2.6 (1)	Nursing students (1)	NR
Extraocular movements examination	2.6 (1)	Nursing Students (1)	NR
Corneal light reflex	2.6 (1)	Nursing students (1)	NR
Cover-uncover test	2.6 (1)	Nursing students (1)	NR
Teller acuity chart	2.6 (1)	Ophthalmologist (1)	NR
Occluder	2.6 (1)	Community healthcare workers (1)	NR
Randot Stereo Test (Stereo Optical)	2.6 (1)	Community healthcare workers (1)	NR
Denver Eye Screening Test	2.6 (1)	Nursing students (1)	NR
Pupillary light reflex	2.6 (1)	Nursing students (1)	NR
Ophtalmoscopic examination	2.6 (1)	Eye technicians (1) Doctors (1)	NR
Tonometry	2.6 (1)	Eye technicians (1) Doctors (1)	NR

* Test types shown as 38 as several studies used more than one type of test.

NR, Not reported.

### Hearing screening

Pure tone audiometry was used most commonly (46.9%), with a sub-group of 21.9% using smartphone/tablet-based audiometry (Table 4). CHWs and nurses were the most common screeners (78.9%; 15/19) with 26.3 and 57.9% of studies using CHWs and nurses (including student nurses), respectively. Almost two thirds of studies (12/19) utilized the same facilitator for vision and hearing screenings, including CHWs, nurses (including nursing students), trained interviewers, and doctors. Referral criteria considered specific threshold levels at frequencies ranging from 0.25 to 8 kHz (Table 4). For children, threshold referral criteria ranged from 25–30 dB at low to mid frequencies (0.25, 0.5, 1 kHz) and from 20–50 dB at mid to high frequencies (1 to 4 kHz). The combined results in Table 4 show that most studies referred children with thresholds >25 dB at two or more frequencies. Two studies specified referral criteria of a pure tone average (>30 dB; ≥41 dB) for adults. Tests specifically conducted on newborns included Transient Evoked Otoacoustic Emissions (TEOAE) and Automated Auditory Brainstem Responses (AABR). A “refer” result from both ears was considered a referral for further testing for both tests (34). Distortion Product Otoacoustic Emissions (DPOAE) was used in only one study with preschool children (35). One study included an otoscopic examination (including video-otoscopy) and tympanometry as part of the primary hearing screening procedures (36). Otoscopy findings of outer or middle ear pathology (e.g., wax impaction, discharge, eardrum perforation) and type B and type C tympanograms warranted referral for further evaluation.

**Table 4:** Hearing screening test type (*n* = 32), facilitators, and referral criteria used across reported studies (*n* = 19).

Type of test: Hearing screening^*^	**% (*n*)**	**Facilitator type (*n*)**	**Referral criteria**
Pure tone audiometry (PTA)	25.0 (8)	Nurse (5) Community healthcare workers (2) Hearing technicians (1) Doctors (1) Medical students (1) Trained volunteers (1) Hearing consultant (1) Audiologist (1) Nursing students (1)	*Children:* Two or more thresholds > 20 dB at 1–8 kHz, > 25 dB at 250–500 Hz (*n* = 2) Threshold > 20 dB at 0.5–8 kHz, > 25 dB at 250 Hz (*n* = 1) Threshold > 20 dB (no frequency specified) (*n* = 1) Threshold > 25 dB at 1 and 4 kHz (*n* = 1) Threshold > 40 dB at 1 kHz in one or both ears (*n* = 1) Threshold > 50 dB at 1 and 4 kHz (*n* = 1) *Adults:* Pure tone average (0.5, 1, 2 kHz) > 30 dB (*n* = 1)
Pure tone audiometry (PTA) (smartphone/tablet-based)	21.9 (7)	Community healthcare workers (4)	*Children:* Thresholds > 25 dB HL at 2 or more frequencies (1, 2, 4 kHz) (*n* = 2) Thresholds > 30 dB HL at 1 kHz and > 25 dB HL at 2 and 4 kHz (*n* = 1) *Adults:* Pure tone average (0.5, 1, 2, 4 kHz) ≥ 41 dB (*n* = 1)
Otoscopy (including video-otoscopy) *Part of primary procedure n = 2* *Supplementary procedure n = 4*	18.8 (6)	Community healthcare workers (4) Nursing students (2)	*Children:* Ear scarring, wax impaction, fluid visible behind eardrum (*n* = 1) Discharge, eardrum perforation, grommets (*n* = 1)
Tympanometry *Part of primary procedure n = 1* *Supplementary procedure n = 2*	9.4 (3)	Nursing students (1) Doctor (1) Community healthcare worker (1)	*Children:* Type B or C tympanogram (*n* = 1)
Transient Evoked Otoacoustic Emissions (TEOAE)	3.1 (1)	Nurses (1) Technicians (1) Pediatricians (1)	*Newborns:* “Refer” result from both ears (*n* = 1)
Automated Auditory Brainstem Responses (AABR)	3.1 (1)	Nurses (1) Technicians (1) Pediatricians (1)	*Newborns:* “Refer” result from both ears (*n* = 1)
Distortion Product Otoacoustic Emissions (DPOAE)	3.1 (1)	Doctor (1)	*Children:* “Fail” result (not specified for 1 or both ears) (*n* = 1)
Spanish Hearing Impairment Inventory for the Elderly (SHIIE)	3.1 (1)	Trained and standardized interviewers (1)	*Elderly adults:* Score ≥ 10 (*n* = 1)
Finger rub test	3.1 (1)	Nurses (1)	NR
Parental Questionnaire	3.1 (1)	Nursing students (1)	NR
Pilot Audiometer (spondaic words)	3.1 (1)	Nursing students (1)	NR
Brainstem auditory evoked response (BERA)	3.1 (1)	Technician (1)	NR

^*^Test types shown as 32 as several studies used more than one type of test.

dB, decibel; kHz, kilohertz; NR, Not reported.

### Reported outcomes

A range of outcomes were reported in the included studies. Referral rates after the initial screening were the most commonly reported outcome with 52.6% of the studies (10/19) specifying this outcome (Table 5). Hearing screening presented higher referral rates compared to vision screening for children and adult populations. Referral rates ranged from 0.48 to 15.7% and 1.6 to 25.4% for vision and hearing screening in children, respectively. Only one study reported referral rates for adult screening. Follow-up rates were reported for only 22.2 and 27.8% of vision and hearing screening studies, respectively. Outcomes of mean test duration and cost were seldom reported.

**Table 5:** Reported program outcomes for combined vision and hearing screening (*n* = 19).

**Reported outcomes**	**Vision screening (*n*)**	**Hearing screening (*n*)**
**Referral rates: initial screen**	10 studies	10 studies
Range: children	0.48–15.7 % (9)	1.6–25.4 % (9)
	Combined vision and hearing screening: 3.8% (1)	
Range: adults	16.8% (1)	69% (1)
**Follow-up rates**	4 studies	5 studies
Range: children	25.1–88.1% (3)	32.5–94.6 % (4)
Range: adults	5% (1)	17.5 % (1)
**Test duration**	3 studies	4 studies
Range: children	91.8–111.0 seconds average (±51.9–60.5 SD)	66.8–105.1 seconds average (±62.3–102.5 SD) and 300 seconds average
	Mean duration for combined vision and hearing screening: 158.6–521.2 seconds (± 85.9–453.8 SD) (2)	
Adults: median time	4 mins	16.7 mins
	Median time for combined vision and hearing screening: 20.5 mins (1)	
**Cost: combined screening**	4 studies	
Range: children	36 cents per child (in the year 1979) $5.63–$6.67 per child (2019, 2021)	
Adults: average cost	For population-based surveys combined screening 11% cheaper	

### Benefits and challenges of combined vision and hearing screenings

Tables 6, 7 summarize the reported benefit and challenges of combined vision and hearing screening programs, respectively. Benefits most commonly reported included early detection, cost and time efficiency, and positive outcomes with community-based screenings. At the same time, poor follow-up, loss to follow-up, and over-referrals were the most common challenges.

**Table 6:** Reported benefits of combined vision and hearing screening.

**Reported benefits**	**References**
Earlier detection of vision and hearing difficulties •Support development and academic performance in children •Support functioning and quality of life in adults	(11, 20, 37, 38)
Cost- and time-efficient relative to screening for hearing and vision separately; benefit to public health services	(39–41)
Community-based service-delivery model •*Greater accessibility to screening services, especially in lower income or resource poor-communities* •*Efficient: combined screening by same tester; CHW or LHW understands context, culture, and language* •*Low-cost (non-specialist personnel)* •*Decentralized service delivery improves follow-up rates and cost-saving for parents/caregivers (less traveling, a day off from work)*	(11, 20, 36, 37, 42)
Enabling mHealth technology •*Efficient: combined screening conducted on the same device by the same tester* •*Enables task shifting with minimal training* •*Cost-effective: one device for both vision and hearing screening, simple technology* •*Cloud-based paperless data management* •*Increased access in lower income or resource-poor communities*	(11, 20, 37)

CHW, Community Health Worker; LHW, Lay health worker.

**Table 7:** Reported challenges of combined vision and hearing screening.

**Reported challenges**	**References**
Poor follow-up/loss to follow-up: •*Families moving away* •*Change in contact numbers of parents/caregivers* •*Lack of staff/facilitators to conduct follow-up assessments* •*School-based screening: child is absent on day of testing* •*Costs involved for parents/caregivers to attend follow-up appointments (traveling, food, away from work)* •*Services not accessible (transport not available)*	(20, 38, 42–44)
Over-referrals for follow-up evaluation increase burden on resource-constrained health services	(45, 46)
Young children may be unable to cooperate	(47)
Lack of clarity of referral and treatment regimens	(47)
Annual screening can be time-consuming	(47)
Special considerations/adjustments needed for difficult-to-test adults (e.g., older adults with cognitive impairment)	(48)
Community-based service-delivery model •*Noise levels affect hearing screening (requires adaptations)* •*Safety in community* •*Safety of equipment* •*Language-diversity*	(11)
Use of mHealth devices for combined screening •*Safety of equipment* •*Charging of equipment* •*Technology: training and retraining is needed*	(11)

## Discussion

This scoping review identified 19 studies reporting combined hearing and vision screening programs of which almost half (9/19) were published in the past 8 years. Key findings are discussed below.

### Target populations

Children (0–18 years of age) were most commonly screened in the included studies (15/19) with most focussed on children aged 3–7 years (10/19) entering pre-primary and primary education. The first three to four years of life are critical for optimal neuroplasticity for cognitive development, including the sensory systems (49, 50). Therefore, combined screening programs can facilitate early detection and intervention of hearing and vision impairments to support early childhood development with long-term benefits for socio-emotional, academic, and vocational outcomes (6, 11, 20, 37, 51–55).

Few studies involved adults (*n* = 4), with an overall age range of 50 to 93 years. The case for population-based adult screening is still unclear as the United States Preventative Service Task Force (USPST) indicated a lack of sufficient evidence regarding hearing screening in asymptomatic adults aged 50 years or older (56). Although annual screening can be time-consuming and costly (47), the WHO recommends periodic or systematic hearing screening for all adults from the age of 50 years (57). Guiding principles and guidelines for hearing screening in adults include target groups, age for screening and frequency (i.e., all adults, 50+ years screened at 5-year intervals moving to 3-year intervals from 65 years of age), settings for screening (i.e., clinical, community, and home settings), screening personnel, screening tests, follow-up, diagnostic assessment, and intervention (57). A recent epidemiological study in the US suggested that adults of ~30 years of age should receive hearing screening as primary prevention, with screening from 45 years of age for secondary prevention (58). Furthermore, research in vision impairment is mostly conducted on adults over 50 years since 80% of vision impairments are found in this age group (1). However, younger adults may also be at risk for vision impairments related to refractive errors and diabetic retinopathy commonly occur much earlier in adults (1). Combined vision and hearing screening for younger adults should therefore also be considered in light of emerging evidence.

Benefits of combined screening for adults include earlier detection and subsequent provision of rehabilitative intervention that can improve their participation in activities of daily living, overall wellbeing, and quality of life (6, 54). The substantial overlap between vision and hearing impairment prevalence, especially for adults 50 years and older, strengthens the rationale for combined screening (19). In addition, combined screening can be cost- and time efficient compared to conducting separate screenings. Only three studies indicated the time efficiency of a combined approach (11, 19, 20). For preschool and school-aged children, combined screening can be completed within ~3–8 mins (11, 20). The time duration for combined screening in adults was specified by only one study, with a median time for both tests of ~20 mins (19). The longer duration compared to childhood screening is attributable to the adult hearing screening determining thresholds compared to a fixed intensity screening in children (19). Nevertheless, with this combined approach, it was possible to test 30 individuals daily (19). Only Bright and colleagues (19) demonstrated the potential cost-benefit of separate vs. combined screening as part of population-based surveys for hearing and vision. The economic benefit of combined screening can also impact public health services as unnecessary referrals and/or follow-up appointments can be reduced (37, 40).

### Type of tests

The most commonly used screening tests were pure tone audiometry for hearing and the Snellen Chart or Tumbling E for vision. Although these tests were most commonly reported, various procedures and referral criteria were implemented across studies limiting comparability. Future research and consensus groups should develop universal guidelines covering procedures and referral criteria such as those stipulated in the hearing screening handbook released by the WHO in 2021 (57). However, such guidelines should be contextualized within countries and integrated with guidelines on vision screenings. Additionally, novel, self-administered tests for hearing and vision screening are also freely available to the general public as smartphone applications, e.g., hearWHO (59) and Peek Acuity (60). Using such self-administered tests *via* mobile application eliminates the need for special equipment and trained personnel, improving access to screening and reducing costs.

### Settings for screenings

All studies involving adults were conducted in home-based settings, facilitated mainly by healthcare professionals (e.g., nurses, doctors, and audiologists) [e.g., (43, 48, 61)]. Most studies involving children were conducted in school settings (11, 20, 36, 37, 39, 41, 42, 45–47). These studies demonstrate the potential for decentralized combined hearing and vision screening in adults and children using community-based service delivery models. Community-based models offer greater accessibility, especially in lower income or resource-constrained communities where access to such services might otherwise be unattainable (11, 20, 37, 42), and demonstrate improved follow-up rates (37). Furthermore, a decentralized service also supports economic benefit to community members and parents/caregivers of infants and children due to limited travel costs and less time from work (36). However, some challenges specific to conducting screening in community settings should be noted, including noise levels affecting hearing screening results, equipment safety, and language barriers (11). Mitigation strategies to minimize the effect of background noise on the outcomes of hearing screening have been suggested, e.g., screening at a higher hearing level at lower frequencies (11, 45). The use of mHealth technologies for hearing screening with automated noise monitoring algorithms also assist with quality control, as used in studies conducted in decentralized settings by Eksteen et al. (11), Bright et al. (19), and Manus et al. (20). Using lay health workers (LHWs) or CHWs from a local community as screening personnel, who understand a specific community's context, culture, and language, can support effective programs (11).

### Screening personnel

The included studies were mainly facilitated by CHWs (31.6 and 26.3% for vision and hearing screening tests, respectively) and nurses (47.4 and 57.9% for vision and hearing screening procedures, respectively). Using CHWs to facilitate combined sensory screenings demonstrates potential, with the WHO recommending task-shifting as a priority to improve access to healthcare service delivery (2). A recent scoping review illustrated the success of task-sharing with CHWs who effectively facilitated screening procedures and encouraged the attendance of community-based hearing screening programs (28). The engagement of CHWs in assessing and addressing hearing healthcare in communities that suffer from disparities in access to care can be an effective way to tailor healthcare strategies to a community's characteristics (62). Vision screening in children conducted by CHWs in resource-poor communities was also effective (63). Training of non-professional personnel prior to conducting screening is an important factor that can affect the outcomes of the screening. The training that non-professionals received was reported in a few studies and it ranged from basic, brief descriptions [e.g., “…volunteers received training…” (39); “…health worker with advanced hearing-health training and extensive experience in ear health…” (36)] to more extensive reports of theoretical and practical training, and follow-up sessions provided (e.g., 11, 20). Therefore, the current review demonstrates the value of using trained non-professionals to improve availability, accessibility, and cost-effectiveness of combined vision and hearing screenings in low-middle income, underserved, and resource-poor communities (e.g., 11, 19, 20, 39). The impact of training of non-professional personnel should be explored in future studies. CHWs can also play a role in raising awareness of the importance of eye and hearing care across the life span, and of preventable causes of hearing and vision impairment (e.g., infectious diseases such as measles, perinatal diseases, nutrition-related diseases, unsafe traditional medicines etc.) (1, 2, 64), and encouraging community members to attend and participate in the screening process.

### Use of mHealth technologies

mHealth technologies for combined sensory screening were also identified as an important enabler for task-shifting, decentralized access, and data management (2). Three articles in this current review used mHealth technologies for the combined screening program (11, 20, 37). Simple user interfaces, automated testing and interpretation, rigorous quality control, and paperless data management are key features supporting use in communities (11, 26, 65). Findings of this review indicate that mHealth technologies require minimal training and allow CHWs or LWHs to facilitate combined sensory screening (11, 20, 37). Healthcare system efficiency can also be improved by mHealth solutions (66, 67). Earlier studies reported over-referrals for follow-up assessments with an increased burden on resource-constrained public health services as a challenge of combined vision and hearing screening (45, 46). However, the use of mHealth technologies within community-based service delivery models has proven effective and scalable, with selective and more appropriate referrals helping to reduce the burden on healthcare systems and scarce specialized healthcare professionals (11, 20, 37). Combined vision and hearing screening using mHealth technologies have also been demonstrated as cost-effective (between $5.63 to $6.67 for full-cost sensory screening per child) (11, 20, 37).

### Challenges to combined screening

Several challenges have been reported for combined vision and hearing screening programs (see Table 7). Deal and Lin (68) reported that most screening studies do not report on adverse outcomes of screening, e.g., failure to receive or attend follow-up services and intervention after a positive screening test, and they identified this as a critical research gap. This highlights the most commonly reported challenge found in this review: the loss to follow-up. Numerous reasons have been identified that result in poor follow-up, as listed in Table 7. Therefore, future studies should carefully consider the setting where combined screening is to be conducted to ensure close monitoring and control of the follow-up system. Eksteen and colleagues (19) reported mitigation strategies for challenges related to a mHealth-supported community-based combined screening program. These included suggestions to improve follow-up attendance, screening equipment maintenance, supervision, and support to trained CWHs or LHWs.

### Limitations and future directions

This scoping review presented some limitations. Although the search was conducted across three databases, gray literature was not included. Only studies published in English were included. Therefore, some relevant work might potentially have been excluded. Given the nature of scoping review, a critical appraisal of studies was not undertaken, and hence no comments on the quality of the included studies are provided. A lack of clarity in referral and treatment regimens was reported as a challenge of combined vision and hearing screening programs (47). Therefore, more research is needed to develop universal, standardized guidelines on screening procedures and referral criteria to support the integration of hearing and eye care globally. Based on the lack of consistent reporting across studies in specific areas related to the combined screening program, a guideline of recommended outcomes to report is outlined in Table 8. Future studies should consider reporting on the recommended outcomes to ensure homogeneity in research methods and reporting format. Standardized reporting will support improved monitoring of combined screening programs and standardize reporting of future evidence (69).

**Table 8:** Checklist with recommended outcomes to be reported in future combined vision and hearing screening studies.

**Recommended outcome to be reported**	**Motivation**
✓ Target disorder and population group	Guide the appropriate selection of screening procedures and referral criteria. Reduce the burden of disease on the population groups most affected.
✓ Screening tests, test procedures, referral criteria	Based on universal guidelines to ensure quality and appropriateness, e.g., WHO Hearing screening recommendations (2021).
✓ Setting (context) and personnel	Monitoring and control of referrals and follow-ups
✓ Referral rates	Determine the percentage of the population referred for further testing.
✓ Follow-up rates	Monitor attendance of follow-up screening or diagnostic testing of those who failed screening.
✓ Feasibility of implementation (e.g., test duration, costs)	Determine feasibility in terms of cost- and time-effectiveness.
✓ Sensitivity and specificity	Evaluate the performance of screening tests.
✓ Positive and negative predictive values	Indicate the likelihood that the screening participant has/does not have the condition that screening targets when the test is positive/negative (positive predictive value/negative predictive value).
✓ Training, monitoring, and supervision of non-professionals	Ensure the effectiveness of the screening program and quality control.

Future studies should also focus on the feasibility and implementation of combined screening programs across different age groups and high-risk populations, and especially in low- and middle-income countries. Only one study reported on combined sensory screening of adults with cognitive impairment, but there is no evidence on combined screening programs for difficult-to-test pediatric populations. Therefore, future studies should also explore special considerations needed for difficult-to-test populations (e.g., children or adults with cognitive impairment).

## Conclusions

The global prevalence of hearing and vision impairment and associated adverse effects emphasizes the need for population-based screening, especially for those most at risk. Even though the population groups most affected by ear and hearing problems are the same as those suffering from the highest burden of vision problems, limited research has been reported on combined hearing and vision screening programs. Studies varied greatly regarding contexts, personnel, screening tests, and reported outcomes. Significant potential and benefit are demonstrated, especially using mHealth technologies for screening and data management within a community-based service delivery model to provide effective, accessible, and affordable combined screening services. However, more feasibility and implementation research are required, particularly in low- and middle-income countries and across all age groups. Universal, standardized reporting guidelines for combined sensory screening programs are required to further improve the standardization and effectiveness to capitalize on the benefits of combined screening programs.

## Author contributions

Establishment of research question(s) and development of search strategy, discussion, and conclusions: IO, CF, VM, SC, and DS. Background framing: SC and DS. Database search and record screening: IO and CF. Extraction of primary studies from the included reviews: IO, CF, and DS. All authors approved the manuscript and its submission to *Frontiers in Public Health* and contributed to the design of the work, discussed the results, and commented on the manuscript.
